# Takotsubo syndrome is triggered by hypoactive delirium and recognized by increased catecholamine requirement in the ICU

**DOI:** 10.1016/j.clinsp.2024.100466

**Published:** 2024-08-08

**Authors:** Carla Alexandra Scorza, Josef Finsterer, Fulvio Alexandre Scorza

**Affiliations:** a Disciplina de Neurociência, Universidade Federal de São Paulo/Escola Paulista de Medicina (UNIFESP/EPM), São Paulo, SP, Brazil; b Neurology and Neurophysiology Center, Vienna, Austria

Delirium is an organic disease characterized by acute and usually temporary disorientation, impaired attention and concentration, confusion, and hallucinations.^1^ Delirium is caused by fever, poisoning (e.g., opioids), illicit drugs, infections, sepsis, dehydration, liver failure, or renal insufficiency.^1^ Delirium can be hyperkinetic or hypokinetic (hypoactive, quiet), in the latter case, patients are lethargic and hypoalert.^2^ Hypoactive delirium can be easily overlooked. In contrast to dementia, in which memory is predominantly impaired, attention deficit is the predominant feature of delirium.^2^

Hyperactive delirium has been repeatedly reported to trigger stress cardiomyopathy, also known as Takotsubo Syndrome (TTS), or broken heart syndrome.^3^^,^^4^ TTS is a transient cardiomyopathy characterized by precordial anginal chest pain, the elevation of troponin and Creatine-Kinase (CK), ST-elevation or depression on Electrocardiogram (ECG), and regional hypokinesia, akinesia, or dyskinesia in the left ventricular myocardium in the absence of significant coronary artery stenosis or occlusion. There are four subtypes of TTS, which are usually diagnosed according to the Mayo Clinic criteria.^5^ To our knowledge, TTS caused by hypoactive delirium has not been reported.

The patient was a 60-year-old female, 160 cm tall and weighing 75 kg, who was admitted for chemotherapy of Diffuse, Large B-Cell Lymphoma (DLBCL) together with mantle cell lymphoma Ann Arbor stadium IVa without MYC rearrangement infiltrating the supra- and infra-diaphragmatic lymph nodes, the right humerus, right scapula, T12 vertebra, L1 vertebra, left os ileum, upper pubic branch, right femoral neck, right femur, the lungs, spleen, and the gluteus muscle. Intravenous steroids were started six days before admission. Concomitant oral hydromorphone was administered for bone pain but had to be switched to transdermal fentanyl two days before admission because of confusion, drowsiness, and dysphagia. Upon admission, the patient could not be contacted and did not follow instructions. She was drowsy, tachycardic (130 min), tachypneic (25 min), and required oxygen (6 L/min). There were elevated CRP, calcium, and uric acid levels. Her medical history was positive for arterial hypertension, diabetes, hyperlipidemia, high-grade left carotid artery stenosis, erysipelas, and psoriasis.

On hospital day 2 (hd2), the patient required intubation, mechanical ventilation and hemodiafiltration due to respiratory insufficiency and acute renal failure. Under these circumstances, she received chemotherapy with cyclophosphamide, rituximab and denosumab with consecutive aplasia and a beneficial effect on lymphoma biomarkers. As she progressed, she developed an increased need for noradrenalin, which is why vasopressin was added to hd3. At hd14, there was a sudden hemodynamic deterioration requiring a significant further increase of norepinephrine and vasopressin. ECG showed ST-depression in the left lateral leads and bedside echocardiography showed decreased left ventricular (LV) systolic function (EF: 37%), and apical akinesia/hypokinesia with apical ballooning. CK-MB and troponin were increased. Classic-type TTS was suspected, and coronary angiography was scheduled. Subsequent repeat echocardiograms showed steady recovery of systolic dysfunction until normalization at six weeks. Coronary angiography showed only mild atherosclerosis without significant stenosis. The patient was successfully weaned and extubated on hd16, but she remained unresponsive to external stimuli. One day after discharge from the ICU, the psychiatrist diagnosed hypoactive delirium and prescribed quetiapine, followed by haloperidol and benzodiazepines. The neurological examination revealed quadriplegia, but the MRI, EEG and cerebrospinal fluid examinations were inconclusive.

The patient presented is interesting in two aspects, TTS developed during mechanical ventilation and was detected with a sudden increase in catecholamine demand and because hypokinetic delirium was missed before intubation, presumably persisted during mechanical ventilation, and most likely triggered the TTS. The diagnosis of TTS in a ventilated patient is difficult as no symptoms are reported and only instrumental findings (rise in troponin, CK, CK-MB, proBNP, infarct ECG, systolic dysfunction) are available when thinking about it. To our knowledge, a sudden increase in catecholamine demand has never been reported as an indicator of TTS. Other triggers of TTS besides delirium, such as stress from malignancy, chemotherapy, mechanical ventilation, hemodiafiltration, anxiety, or pain from bone infiltration, were considered but discarded because these conditions occur frequently without ever triggering TTS and because the patient received adequate analgesia and sedation to reduce stress.

The most plausible trigger for delirium in the index patient was the combination of steroids and opiates. It is known that steroids can occasionally cause delirium.^6^ However, there are also studies indicating that steroids are generally safe in terms of causing delirium.^7^^,^^8^ It is also known that opiates can cause delirium, although the incidence of delirium in previously opiate-naïve patients does not differ between opiate types.^9^ Whether the derailment of diabetes with diabetic encephalopathy contributed to the development of delirium, remains speculative. Because delirium was most likely present before intubation, there was no cerebral involvement in lymphoma, and there was no history of alcoholism, the combination of steroids and opiates remained the most plausible cause.

This case shows that hypokinetic delirium can trigger TTS and that a sudden increase in catecholamine demand during mechanical ventilation can be an indication of TTS. In patients with confusion, attention deficit and impaired consciousness, an immediate, thorough neurological and psychiatric examination is required to avoid overlooking hypokinetic delirium, which can have a strong impact on the course of the disease.

## Compliance with ethics guidelines

This article is based on previously conducted studies and does not contain any new studies with human participants or animals performed by any of the authors.

## Authors’ contributions

JF: Design, literature search, discussion, first draft, critical comments, final approval. CS and FS: literature search, discussion, final review.

## Funding

No funding was received.

## Declaration of competing interest

The author declares that the research was conducted in the absence of any commercial or financial relationships that could be construed as a potential conflict of interest.

## Data Availability

Data that support the findings of the study are available from the corresponding author.
